# Supplementary material for: SUITPy: A Python-based toolbox for the analysis of cerebellar functional and anatomical imaging data across the human lifespan
Source: Imaging Neurosci (Camb). 2026 Aug 6;4:IMAG.a.1323. doi: 10.1162/IMAG.a.1323 (PMC13449926; doi:10.1162/IMAG.a.1323)
Supplement: Supplementary file 1 [file IMAG.a.1323_supp.pdf]

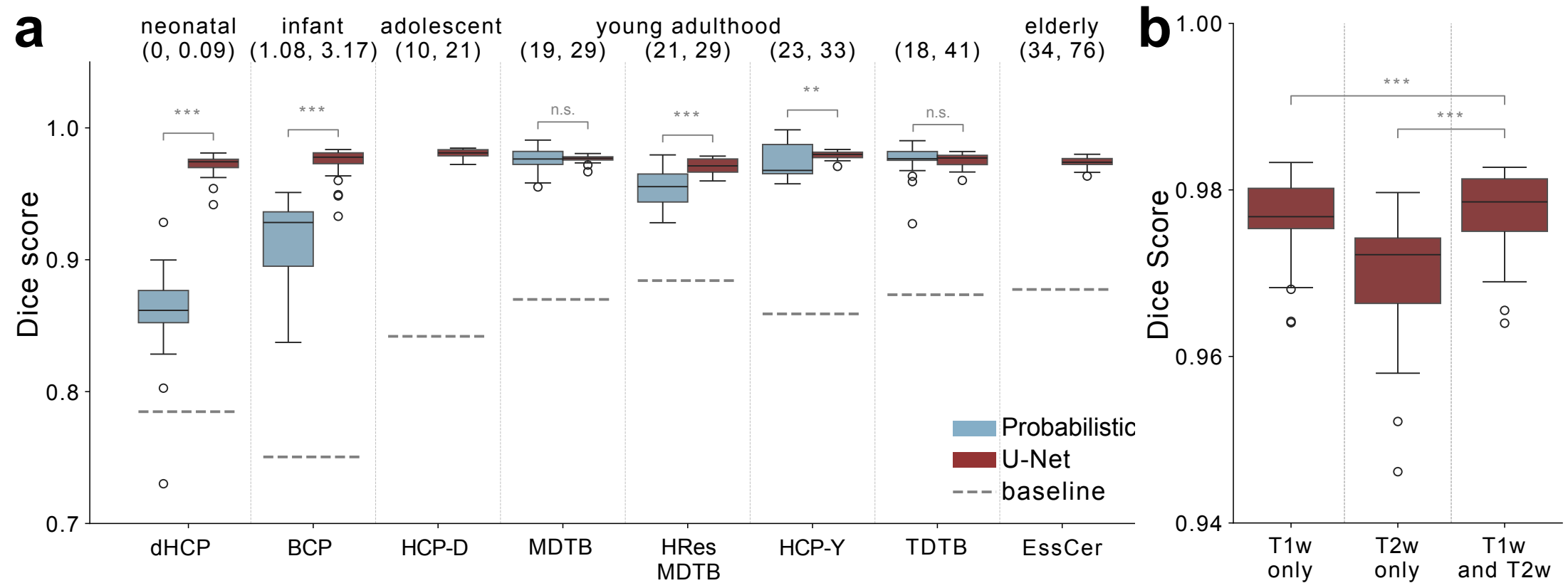

Figure S1. Dice score comparison of U-Net and probabilistic isolation algorithm (a) Dice score for Probabilistic (blue) and U-Net (red) isolation. Dotted lines indicate baseline performance of the simple average of the training set. Horizontal bars with asterisks denote significant pairwise differences (paired t-tests, n.s.: not significant, *: $p<0.05$, **: $p<0.01$, ***: $p<0.001$). The probabilistic isolation failed completely in HCP-D and EssCer datasets, so no pairwise test was executed. **(b)** Dice score comparison of U-Net isolation algorithm using different input modality configurations.

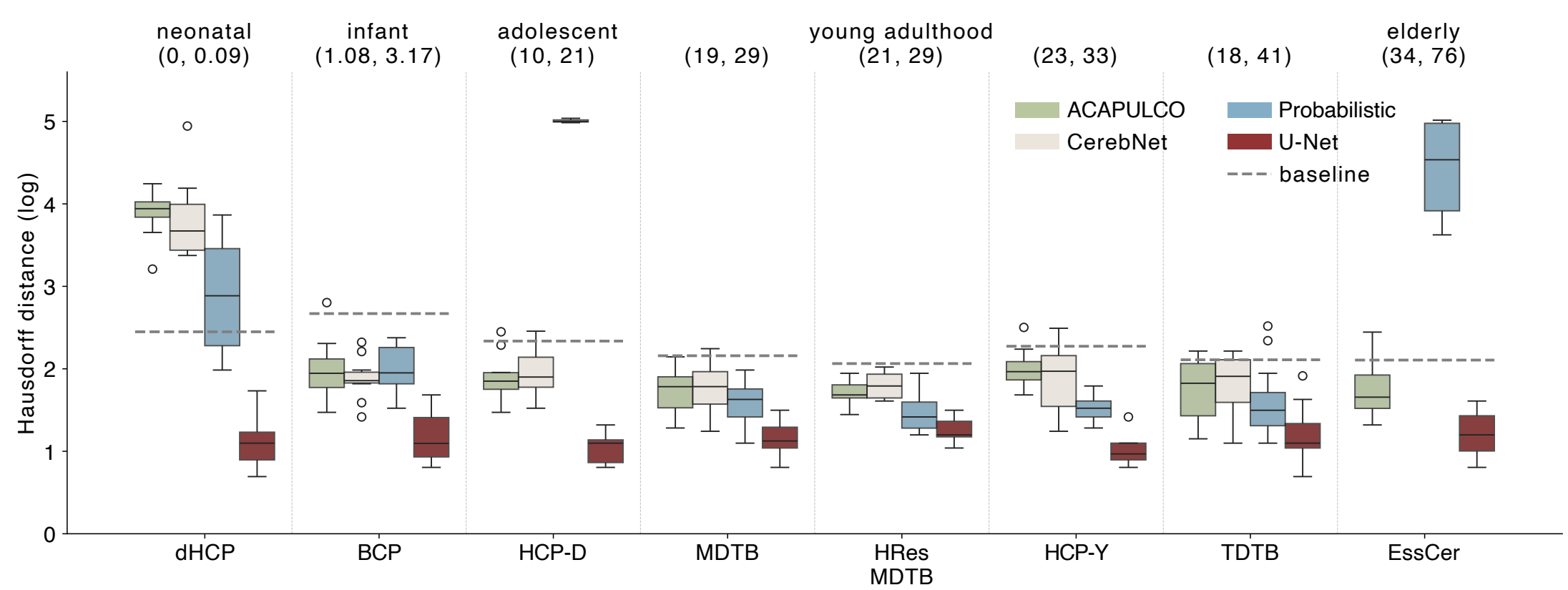

Figure S2. Hausdorff Distance comparison of U-Net with other tools. Dotted lines indicate baseline performance (simple average of the training set). ACAPULCO in beige, CerebNet in sage green, probabilistic isolation is shown in blue, and U-Net isolation in red. For the EssCer dataset, CerebNet results are not shown due to brain segmentation failure.
